# The duplexity of insulin: The integrated bioinformatics analysis and machine learning identified key genes for type 2 diabetes

**DOI:** 10.1016/j.bbrep.2025.102099

**Published:** 2025-06-24

**Authors:** Nan Gao, Xiteng Chen, Jun Yang, Yuanfeng Jiang, Shaochong Bu, Xiaomei Bai, Zhenyu Kou, Chunjun Li, Fang Tian

**Affiliations:** a Tianjin Key Laboratory of Retinal Functions and Diseases, Tianjin Branch of National Clinical Research Center for Ocular Disease, Eye Institute and School of Optometry, Tianjin Medical University Eye Hospital, Tianjin, China; b Department of Endocrinology, Tianjin Union Medical Center, Nankai University, Tianjin, China

**Keywords:** Type 2 diabetes mellitus, Insulin, RNA-Seq, Inflammation, Oxidative stress, Machine learning

## Abstract

**Background:**

Insulin therapy is still the most important treatment for T2DM, but the discussion about whether insulin brings more benefits or harms to T2DM patients has not stopped. Therefore, we used high-throughput RNA sequencing to investigate the role of insulin in T2DM and its molecular changes.

**Method:**

We collected peripheral blood samples from 16 patients with T2DM, and performed RNA-seq on peripheral blood mononuclear cells. Bioinformatics analysis and machine learning were uesd to identify the key differential genes and transcription factor networks. In addition, we performed the flow cytometry and staining to observe ROS level and endothelial-monocyte adhesion in PBMCs of both groups.

**Results:**

A total of 529 differential genes were identified by bioinformatics analysis. 8 genes were identified as key genes, among which IL-6 had high importance in the random forest model. In transcription factor analysis, IL-6, RETN, CTSG and ELANE have abundant transcriptional regulatory relationships. Flow cytometry showed that ROS production, phagocytosis, leukocyte adhesion in insulin treatment group were lower than that in non-insulin treatment group.

**Conclusion:**

Insulin therapy is bidirectional, it can cause islet B cell damage and vascular complications, but also can reduce the level of inflammation and oxidative stress.

## Highlights

• The integrated machine learning and bioinformatics analysis.
•Revealing the two-sided nature of insulin at the genetic level.
•Downregulation of IL-6 and proteasome reduces inflammation and oxidative stress.
•Despite the side effects, insulin therapy was generally favorable.

## 1 Introduction

By 2045, the diabetic population will increase from 463 million in 2019 to 700 million based on the most recent International Diabetes Federation (IDF) report [1]. Patients with diabetes are at high risk of developing chronic systemic complications involving multiple organs, such as neuropathy, retinopathy, kidney disease, and cardiovascular disease. These complications further increase rates of disability and mortality, resulting in a significant public health and economic burden [2,3]. Good glycemic control can prevent or delay the progression of complications of T2DM [4,5]. Although oral hypoglycemic agents have become the first - and second-line treatments for T2DM, a large amount of patients with T2DM still require insulin therapy as the disease progresses [6].

Since its discovery, insulin has been widely used to treat diabetes [7]. For a long time, insulin has been considered as a treatment for the late stage of T2DM. Many studies have indicated that early application of insulin is beneficial in reducing insulin resistance, reversing glucose toxicity, and preserving islet B cell function [[6], [7], [8]]. Nevertheless, long-term high insulin exposure may aggravate insulin resistance and lead to islet B cell failure in T2DM [3]. Monnier and Herman pointed out that high doses of insulin can promote oxidation, which may increase the risk of cardiovascular complications and death in T2DM patients [9,10]. In addition, large dosage of insulin can also lead to obesity, recurrent hypoglycemia, and iatrogenic hyperinsulinemia [9].

PBMCs are the predominant cells involving in the inflammatory process and oxidative stress, play an important role in the progression of T2DM. Previous studies investigated the molecular level changes of PBMCs during the development of T2DM [11,12]. However, the underlying pathogenic mechanism of PBMCs and their interaction with insulin treatment in T2DM have not yet been fully clarified. Given the two-sided nature of insulin, to elucidate the effects of insulin treatment on the regulation of gene expression in type 2 diabetes, we utilized RNA sequencing for the first time to investigate the molecular changes induced by insulin treatment and performed a combined dry-lab and wet-lab methodology [13] to perform a bioinformatics analysis on the transcriptome of PBMCs in patients with Type 2 diabetes.

## 2 Data and methods

### 2.1 Inclusion and exclusion criteria

This retrospective study included 16 patients with T2DM, 8 in the insulin-treated (DM R+) group and 8 in the non-insulin-treated (DM R-) group. Written informed consent was obtained from all participants. Inclusion criteria included: (1) patients with clinically diagnosed T2DM; (2) HbA1c > 6.5 % and <10 %; (3) treatment with either oral hypoglycemic drugs or insulin. Exclusion criteria included: patients with type 1 diabetes, T2DM without treatment, patients using combined therapy with both oral hypoglycemic agents and insulin, patients with malignant tumors, autoimmune diseases or other metabolic diseases and/or long-term use of systemic cortical steroids, patients who were taking medications or supplements that could potentially affect glucose metabolism or cardiovascular function (including those using unknown drugs or health supplements).

### 2.2 Statistical analysis

Clinical data such as gender, age, duration of diabetes, HbA1c level, and diabetic complications were collected. Clinical data were analyzed with IBM SPSS Statistics 25. Categorical variables were presented as frequencies, and continuous variables as means with standard deviations. Normality testing was conducted on the data; if normality was confirmed, parametric testing (independent samples *t*-test) was used to compare means. If normality was not confirmed, non-parametric testing (Mann-Whitney *U* test) was employed. Proportions were compared with Fisher's exact test. Two-tailed tests were used, and P < 0.05 was considered statistically significant.

### 2.3 RNA extraction

After collecting the blood samples, centrifuge them at 2000g for 15 min at a pre-cooled temperature of 4 °C, discard the upper plasma layer, and add 5 mL PBS to the centrifuge tube for dilution. Subsequently, isolate peripheral blood mononuclear cells (PBMC) using 5 mL Ficoll. The centrifuge temperature is adjusted to 20 °C, followed by centrifugation at 751*g* for 20 min. After centrifugation, discard the supernatant and wash with an appropriate amount of PBS, then centrifuge at 751 g for 8 min. Discard the supernatant again and add 1 mL TRIzol to thoroughly lyse the cells. Add 0.2 mL chloroform, vortex vigorously for 15 s, and let stand at room temperature for 3 min. Centrifuge at 12,000 g for 15 min at 4 °C, then carefully aspirate the aqueous phase. Add 0.5 mL isopropanol, invert several times to mix, and incubate at room temperature for 10 min. Centrifuge at 12,000 g for 10 min at 4 °C and discard the supernatant. Wash the pellet with 1 mL of 75 % ethanol, centrifuge at 12,000 g for 5 min at 4 °C, and discard the supernatant. Air-dry the pellet by inverting the tube at room temperature for 5–10 min, then resuspend in 20 μL RNase-free water.

### 2.4 Processing of RNA-seq data

RNA samples will undergo library preparation and transcriptome sequencing using the DNBSEQ-T7 sequencing platform with standard dual-indexed protocols. Raw reads were first separated into FASTQ files of pair-end reads, with FastQC (version 0.11.5) used for data quality control. The clean reads were aligned to the human reference genome (USCS hg19) by HISAT2 (version 2.1.0). Finally, SAMtools (version 1.9) and HTSeq (version 0.6.1p1) were used to quantify and map the reads to an annotated document (GENCODE, version 39lift37, Oct 2021).

### 2.5 Differential gene expression analysis

The R package DESeq2 [14] was then used to identify the DEGs among the DM R+ and DM R-groups. The cutoff criteria for determining DEGs were |log2 fold change (FC)| > 1 and FDR <0.05.

### 2.6 PPI network and identification of hub genes

String database (version 11.5) [15] was used to construct a Protein-Protein Interaction (PPI) network based on the DEGs. We then used cytoscape software to further analyze the protein interaction network [16]. The degree of each protein in the network is calculated by cytoHubba plugin [17], and the top 10 genes are selected as hub genes according to the degree from the largest to the smallest. Molecular Complex Detection (MCODE) plugin was used to identify key protein clustering networks. After that, we will select the genes closely related to diabetes and its complications as the final hub genes.

### 2.7 Machine learning

Random forest (RF) algorithm was used to further screen the key genes in the insulin treatment group. The R package randomForest was used for RF analysis. First, a random seed was set to ensure result reproducibility. The dataset was split into training and testing sets at a 2:1 ratio. In the training set, the number of trees to grow (ntree) and the number of variables randomly sampled as candidates at each split (mtry) were determined through iterative optimization, with 5-fold cross-validation applied to ensure model accuracy. After finalizing the model, predictions were performed on the test set, and the F1-score was calculated to evaluate model performance. Finally, the model will be used to make predictions on all the data. The genes with the top 25 importance in the model and the cross genes in the PPI network are selected as candidate hub genes.

### 2.8 Functional and pathway enrichment analysis

Gene Ontology (GO) functional enrichment analysis [18] was used to explore the function of key protein clustering networks, performed by the clusterProfiler package in R [19]. Then we used gene set variation analysis (GSVA) [20] for pathway enrichment analysis of DEGs, and used limma package in R software to identify the differential pathways between the two groups.

### 2.9 Identification of key transcription factors

We use ChEA3 database [21] to find transcription factors related to hub genes and overlap them with differential genes. Then, according to the ranking in the database, the top 10 were selected as the key transcription factors. We then used cytoscape to construct transcription factors-genes regulatory networks.

### 2.10 Co-expression analysis

Finally, we verified the correlation between genes and between genes and pathways by the co-expression Spearman correlation analysis of hub genes, transcription factors and differential pathways.

### 2.11 ROS assay

DCFH-DA assay was employed to determine intracellular ROS (reactive oxygen species) levels. Freshly isolated PBMCs were treated with 30 μL PBS containing 10 μM DCFH-DA (Sigma–Aldrich, St. Louis, MO, USA) for 30 min at 37 °C in the dark. After the incubation, the cells were rinsed with PBS three times to remove excess DCFH-DA. Intracellular ROS levels were measured under a flow cytometry analysis.

### 2.12 Endothelial-monocyte adhesion assay

HRMECs were seeded in 6-well plates and allowed to reach confluence. Freshly isolated PBMCs were labeled with 2′, 7′-bis (2-carboxyethyl)-5 (6)-carboxy-fluorescein acetoxymethyl ester (BCECF/AM, 10 μM, Thermo Fisher, Waltham, MA, USA) in serum-free RPMI 1640 media for 45 min at 37 °C in the dark. And the labeled PBMCs were incubated with HRMECs for 1 h at 37 °C. And then cells were stained with DAPI and visualized under a fluorescence microscope or resuspended for the DCFCE fluorescent signal using flow cytometry analysis.

### 2.13 Phagocytosis assays

Fluorescein isothiocyanate (FITC)-conjugated dextran (40 kDa, Molecular Probes, Life Technologies, Carlsbad, CA, USA) was used to determine the phagocytic function. Freshly isolated were incubated with 20 mg/L dextran-FITC for 30 min at 37 °C followed by washing three times with 500μL of complete RPMI 1640 medium to remove the free dye. Average fluorescence intensities and the percentages of dextran-positive cells were determined by flow cytometry.

## 3 Results

### 3.1 Patients and data collection

**Fig. 1:**
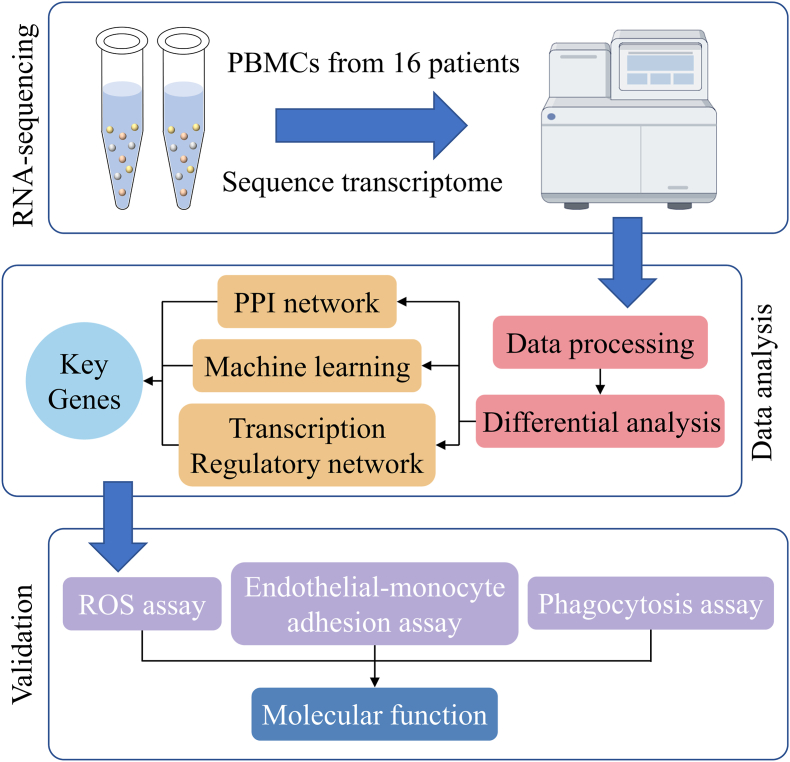
Study flow chart.

The design flowchart of this study is shown in Fig. 1. Table 1 shows the baseline demographic information of patients. Table S1 displays the results of normality testing using the Shapiro-Wilk method; all continuous variables conformed to a normal distribution, an independent samples *t*-test is employed for statistical analysis. There were no significant differences in gender, age, duration of diabetes and HbA1c level between the two groups (P > 0.05). There were no systemic complications of diabetes in these patients.

**Table 1:** Baseline demographic information of patients.

Group	DM R+^a^	DM R-a	P value
n	8	8	
Sex (M/F)	4/4	2/6	0.68^a^
Age (years, mean ± SD)	67.88 ± 9.89	65.38 ± 7.05	0.57^b^
Duration (years, mean ± SD)	15.88 ± 9.34	13.13 ± 9.64	0.57^b^
HbA1c (%, mean ± SD)	7.16 ± 0.65	7.51 ± 1.34	0.52^b^
Race	East Asian (China)	East Asian (China)	
Complications	None	None	

a DM R+ represents the insulin-treated group and DM R− represents the non-insulin-treated group; a: Fisher's exact test; b: Independent-sample *t*-test.

### 3.2 Biological repeat and differential gene expression analysis

In correlation analysis of biological repeat, all samples were highly correlated (R > 0.8) (Fig. S1A and B). We identified 529 DEGs in RNA-seq data according to the cut-off criteria. Volcano plots demonstrating differential expressions are shown in Fig. S1C. Fig. S1D presents the expression heatmap of DEGs.

### 3.3 Identification of hub genes via PPI network

**Fig. 2:**
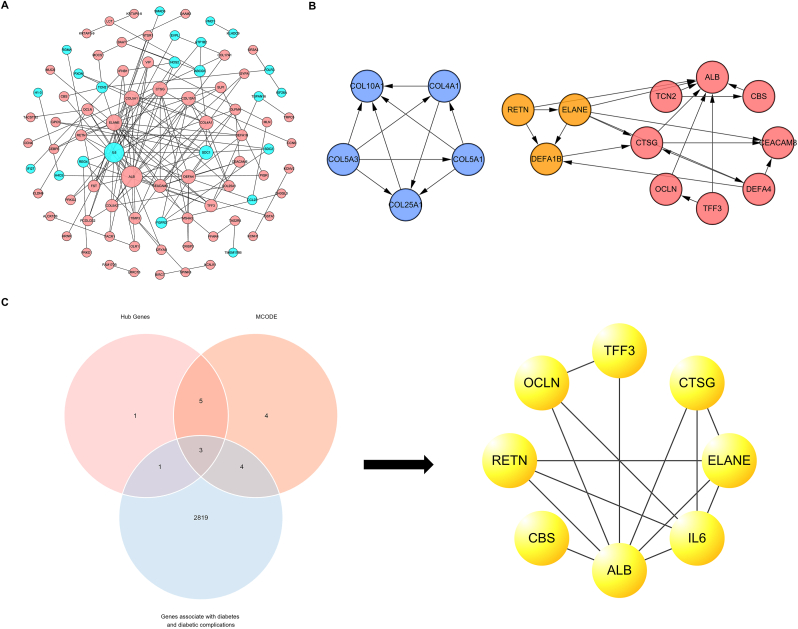
**Protein interaction networks and Hub genes**. (A) The protein-protein interaction network of DEGs. The size of the node indicates the degree of the gene in the network. The higher the degree, the larger the node size. Node color represents gene expression. Red represents up-regulation of gene expression and blue represents down-regulation of gene expression. (B) Key protein clustering networks. The node colors were used to distinguish different clustering networks. Red represents network 1, blue represents network 2, and green represents network 3. (C) Venn diagram. The Venn diagram shows 8 proteins associated with diabetes and its complications in hub genes and key protein clustering network.

All 529 DEGs included 86 proteins and 137 PPI relationships. Fig. 2A shows the PPI network of DEGs. According to the degree calculated by cytohubba, we have identified 10 hub genes, which are ALB, IL-6, ELANE, COL5A1, CTSG, SDC1, COL4A1, COL10A1, DEFA4 and CEACAM8. Fig. 2B shows three key protein clustering networks identified by MCODE. Based on the overlap of hub genes, key protein clustering network and genes associate with diabetes and its complications, we screened 8 genes (IL-6, ALB, CTSG, ELANE, CBS, OCLN, RETN, TFF3) (Fig. 2C).

### 3.4 Identification of hub genes via machine learning

**Fig. 3:**
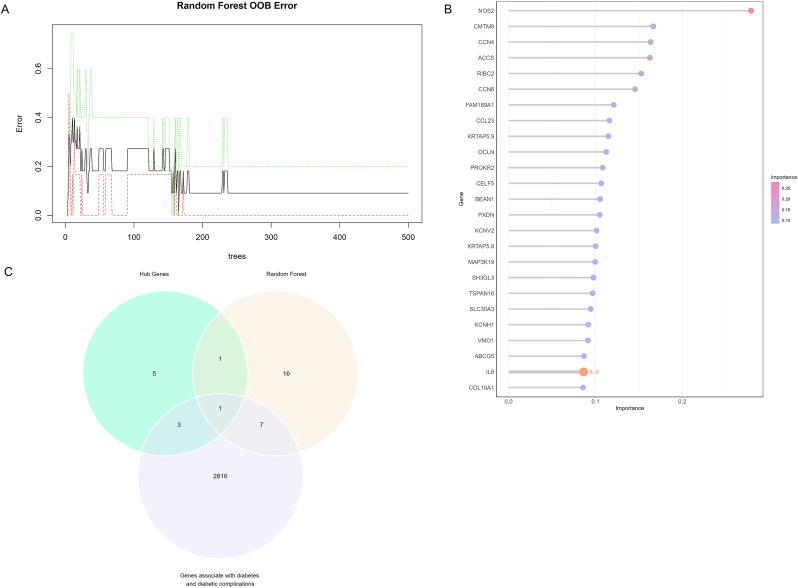
**Machine learning in screening hub genes induced by insulin therapy**. (A) The random forest algorithm shows the error rate. The black curve represents the overall error rate. (B) Lollipop chart. The lollipop chart shows the ranking of DEGs based on the importance score. (C) Venn diagram. According to the Venn diagram, IL6 is a hub gene in the PPI network and a candidate gene screened in the random forest model.

The coding genes in the DEGs were used for RF analysis. Through iterative testing, we determined that the model error is stable when ntree is 500 and the error is minimal when mtry is 4. Using the above parameters to construct the RF model, the final OOB (out-of-bag) error is 9.09 % (Fig. 3A). The F1-score calculated after using the test set for prediction is 1, which indicates that the model predicts well. Fig. 3B illustrates the ranking of genes by the RF model based on the calculation of the importance of each gene. The intersection of the top 25 most important genes from RF and 10 hub genes from the PPI network via venn plots showed that only IL-6 was associated with diabetes and its complications (Fig. 3C). These results indicate that IL-6 plays an important role in the molecular regulation related to insulin.

### 3.5 Functional enrichment analysis

**Fig. 4:**
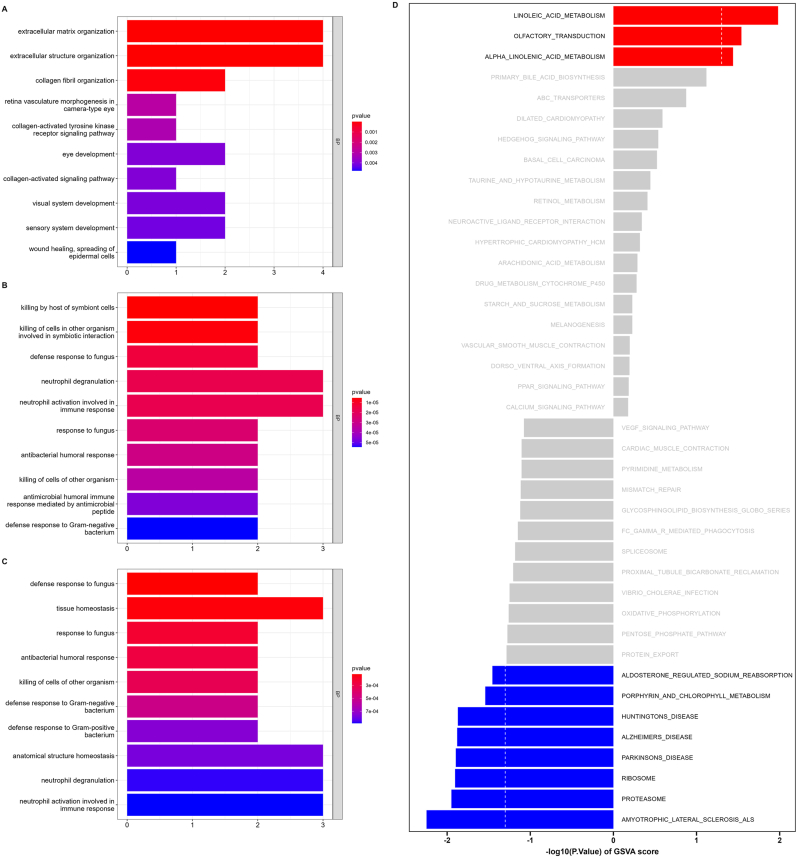
**The result of functional enrichment analysis.** (A–C) The bar plot of top 10 significant GO terms in biological process (BP) of each clustering network. (D) The bar chart of top 40 GSVA enrichment pathway, red indicates the pathway is up-regulated in DM R+ group, blue indicates the pathway is down-regulated in DM R+ group, and gray indicates no difference.

The GO analysis results showed that extracellular matrix organization (GO:0030198, *P*-value = 9.31E-07) was the most significantly enriched biological process in clustering network 1, killing by host of symbiont cells (GO:0051873, *P*-value = 5.47E-06) was the most significantly enriched biological process in clustering network 2, and defense response to fungus (GO:0050832, *P*-value = 0.00013) was the most significantly enriched biological process in clustering network 3. In GSVA analysis, the expression level of ribosome pathway was significantly decreased in R+ group (*P*-value = 0.012, log2FC = −0.54), followed by proteasome pathway (*P*-value = 0.011, log2FC = −0.36), while the expression level of linoleic acid metabolism pathway was significantly increased (*P*-value = 0.01, log2FC = 0.32). The GO analysis results of the key protein clustering network and the GSVA results of DEGs are shown in Fig. 4A–D. Top five biological process of each cluster are shown in Table S2. Table S3 shows the top five differential GSVA enrichment pathways.

### 3.6 Identification of key transcription factors

**Fig. 5:**
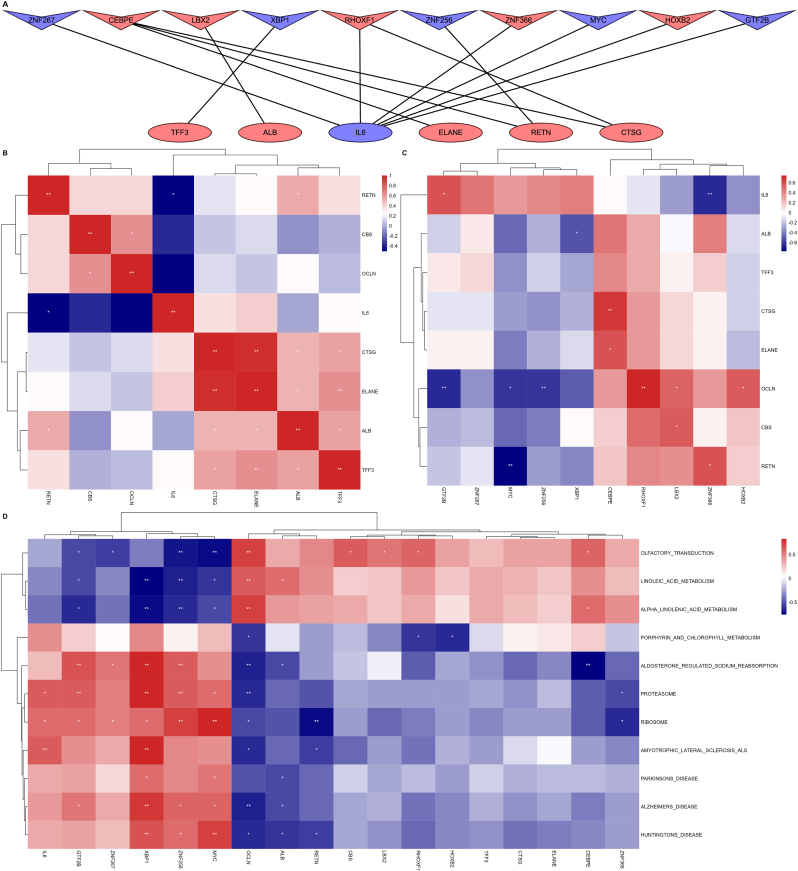
**The regulation network of transcription factors and gene-transcription factor-pathway co-expression heatmap.** Red represents positive correlation and blue represents negative correlation. ∗ Indicates p-value <0.05, ∗∗ indicates p-value <0.01. (A) The regulation network among 10 transcription factors and 6 hub genes. V represents transcription factors and ellipse represents hub genes. (B) Gene-gene co-expression heatmap. (C) Gene-transcription factor co-expression heatmap. (D) Gene-pathway co-expression heatmap.

A total of 1632 transcription factors related to hub genes were identified by ChEA3 database (https://maayanlab.cloud/chea3/), of which 66 were differentially expressed. According to the ranking of transcription factors in the analysis, we selected the top 10 transcription factors as key transcription factors, and they were CEBPE, RHOXF1, ZNF256, GTF2B, ZNF267, ZNF366, XBP1, LBX2, MYC and HOXB2. The key transcription factors and their regulated target genes are shown in Table 2, and the transcription factor regulatory network is shown in Fig. 5A. The results showed that CEBPE regulated the most target genes, and IL-6 as the target gene received the most regulation of transcription factors.

**Table 2:** Top 10 transcription factors and their target genes.

TFs	Rank	Score	Log_2_ Fold Change	Overlapping Genes
CEBPE	4	0.001	1.15	CTSG, RETN, ELANE
RHOXF1	10	0.003	0.93	IL-6, CTSG
ZNF256	30	0.007	−0.53	RETN
GTF2B	76	0.016	−0.54	IL-6
ZNF267	98	0.020	−0.54	IL-6
ZNF366	132	0.027	0.95	IL-6
XBP1	173	0.038	−0.34	TFF3
LBX2	184	0.040	0.40	ALB
MYC	186	0.041	−0.55	IL-6
HOXB2	225	0.051	0.45	IL-6

### 3.7 Co-expression analysis

In gene co-expression analysis, CTSG, ELANE, ALB and TFF3 ad significantly positive co-expression pattern with each other (R > 0.5, *P*-value <0.05), which indicates that there may be a synergistic effect between them. IL-6 and RETN had significantly negative co-expression pattern (R = −0.51, *P*-value = 0.046), this suggests they may have the opposite effect (Fig. 5B). In gene-transcription factor correlation analysis, transcription factors ZNF366 and GTF2B had significantly co-expression pattern with IL-6 (R = −0.63 and 0.57, *P*-value = 0.0086 and 0.021). Transcription factor CEBPE had significantly co-expression pattern with ELANE and CTSG (R = 0.67 and 0.59, *P*-value = 0.0047 and 0.016) (Fig. 5C). In gene-pathway correlation analysis, IL-6, ZNF366 and GTF2B were significantly positive corelated with proteasome and ribosome pathway, while RETN was negatively correlated with proteasome pathway (*P*-values <0.05), this suggests that gene regulation in peripheral blood of DM R+ group is closely related to inflammation, and the use of insulin could reduce the level of peripheral blood inflammation (Fig. 5D).

### 3.8 Downregulated ROS level, leukocyte adhesion and phagocytosis in DM R+ group

**Fig. 6:**
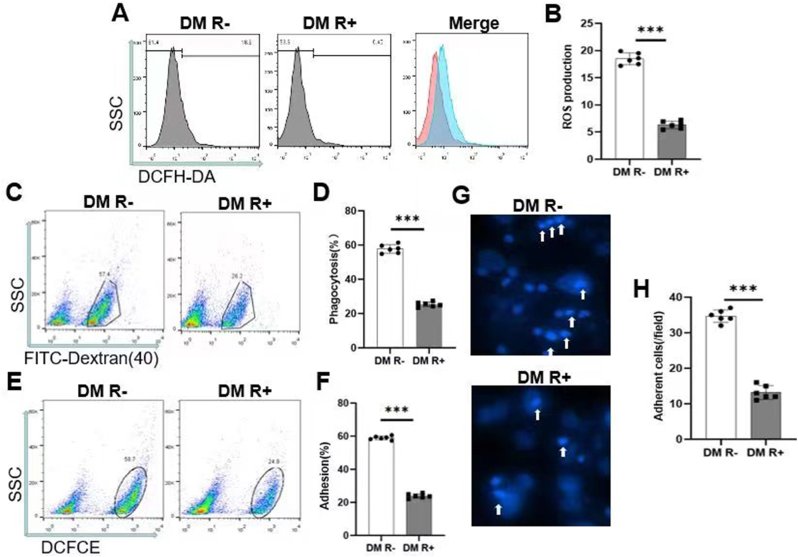
**Downregulated ROS level, leukocyte adhesion and phagocytosis in DM R+ group.** (A) Production abatement of ROS in DM R+ group. (B) Bar chart shows ROS production of each group. (C) The phagocytosis of cells in DM R+ group is weakened. (D) Bar chart shows phagocytosis of each group. (E) leukocyte adhesion is downregulated in DM R+ group. (F) Bar chart shows leukocyte adhesion of each group. (G) Downregulation of adhesion cells in DM R+ group. (H) Bar chart shows adhesion cells of each group. ∗∗∗p < 0.001.

Considering the importance of IL-6 in the previous analysis and its link to inflammation and oxidative stress, we performed experiments to validate its function. To explore the changes of ROS level, phagocytosis and leukocyte adhesion in DM R+ group, flow cytometry and staining were used to detect relevant indicators. Flow cytometry showed that ROS production, phagocytosis, leukocyte adhesion in DM R+ group were lower than that in DM R-group (Fig. 6A–F). DAPI staining showed that the number of adherent cells in DM R+ group was less than that in DM R-group (Fig. 6G and H). The above results are statistically different, indicating that phagocytosis, leukocyte adhesion was significantly reduced in DM R+ group.

## 4 Discussion

In our study, we found that the expression of interleukin-6 (IL-6) was significantly decreased in the DM R+ group, suggesting that insulin may alleviate inflammation in T2DM patients. IL-6 is a cytokine with a wide variety of biological functions in immunity, tissue regeneration, and metabolism, which plays an important role in the pathogenesis of T2DM [22].

IL-6 is closely related to insulin resistance. By participating in the regulation of signal molecules such as nuclear factor kappa B (NF-κB), signal transducer and activator of transcription 3 (STAT3), cytokine signaling 3 (SOCS-3) and insulin receptor substrate (IRS), IL-6 promotes oxidative stress and inflammatory responses, leading to impaired biological effects of insulin, thus mediates insulin resistance [22,23]. Recent studies have suggested that only IL-6 in adipocytes has pro-inflammatory and insulin-resistant effects, while IL-6 produced in skeletal muscle has anti-inflammatory and insulin-sensitizing effects [24,25]. Although our study could not distinguish the source of IL-6 in PBMC obtained from the peripheral blood, we have verified that the ROS level in DM R+ group was significantly lower than that in DM R-group, as shown in Fig. 4, which also suggested that the application of insulin may be beneficial for reducing inflammation and accordingly improving insulin resistance [26].

In addition, the proteasome pathway associated with IL-6 was downregulated in the DM R+ group. Ubiquitin-proteasome plays a role in a variety of cellular processes, including DNA damage response, mitochondrial maintenance and mitophagy, lysosomal degradation, T-cell receptor signaling, and NF-κB signaling [27,28]. Proteasome was shown to be involved in the development of T2DM. Balasubramanyam M et al. [29] suggested that SOCS-1 and SOCS-3 cause IRS to be interpreted by the proteasome by mediating IRS ubiquitination, and this abnormal degradation leads to the inhibition of insulin signaling. Protein degradation caused by ubiquitin-proteasome is also associated with various diabetic complications such as diabetic cardiomyopathy, diabetic nephropathy, and diabetic retinopathy [[30], [31], [32]]. The downregulation of the proteasome pathway suggests that insulin therapy may delay the onset of diabetic complications or attenuate their damage to the body, which is consistent with the results of our cell experiments (Fig. 6). Meanwhile, the expression of the IL6 signaling-related transcriptional repressor ZNF366 was upregulated, while the expression of the transcriptional initiator GTF2B was downregulated [33,34]. Correlation analysis further revealed a strong association between their expression patterns, consistent with the downregulation of IL6 expression at the transcriptional level.

**Fig. 7:**
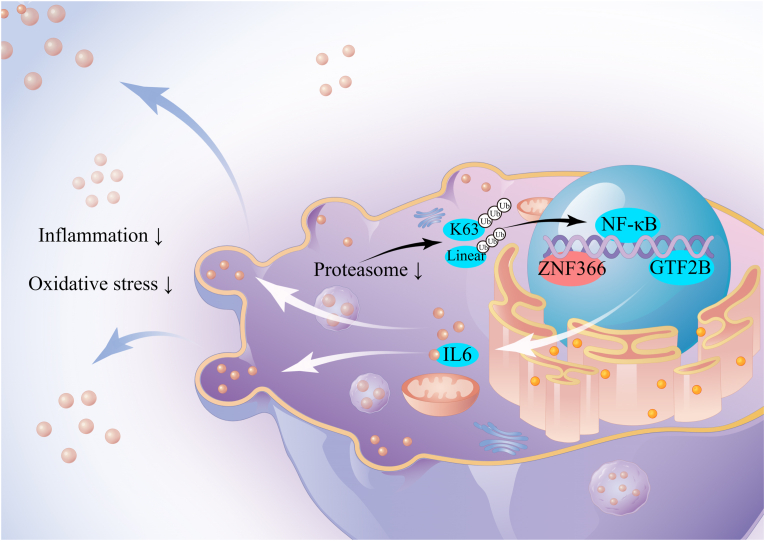
Downregulation of the proteasome inhibits the NK-κB pathway, together with up-regulation of ZNF366 and down-regulation of GTF2B, which jointly inhibit IL-6 production, which is ultimately reflected in the reduction of inflammation and oxidative stress.

These observations led us to hypothesize putative regulatory interactions between IL-6 signaling and proteasome (Fig. 7). We propose that the proteasome may modulate IL-6 production through regulation of the NF-κB pathway. Furthermore, the upregulation of transcriptional repressor ZNF366 coupled with downregulation of transcriptional activator GTF2B could collectively contribute to reduced IL-6 transcription. These coordinated effects might ultimately lead to diminished inflammation and oxidative stress, suggesting a potential mechanism by which insulin treatment could alleviate inflammation in T2DM patients.

The current study revealed that several cytokines—including RETN, CTSG, and ELANE, which can promote vascular damage and insulin resistance—were upregulated in the DM R+ group. Resistin (RETN) is a cytokine produced by adipose tissue involving in glucose and lipid metabolism, which is known to be associated with inflammation and insulin resistance [35,36]. Previous studies showed that RETN could accelerate the process of atherosclerosis by upregulating vascular endothelial adhesion factors such as VCAM-1 and ICAM-1 [35]. Cathepsin G (CTSG) and neutrophil elastase (ELANE), were both found to be secreted by neutrophils and play an important role in promoting inflammatory response and vascular endothelial cell permeability, which was closely related to the pathophysiology of diabetes. In the pathological process of T1DM, CTSG is involved in mediating the activation of CD4^+^ T cells, leading to islet inflammation and apoptosis of islet B cells, thereby affecting the function of islet B cells and leading to the progression of the disease [[37], [38], [39]]. ELANE showed a synergistic effect with CTSG in our results. ELANE, a serine protease secreted by neutrophils, is thought to alter vascular permeability and be involved in vascular leakage, particularly in diabetic retinopathy [40,41]. Up-regulation of these genes may explain why the use of insulin leads to an increased risk of vascular complications. In transcription factor analysis, CEBPE, a transcription enhancer also associated with granulocyte maturation [42,43], was upregulated in the DM R+ group. Correlation analysis revealed that CEBPE expression was significantly positively correlated with CTSG and ELANE levels, further supporting their upregulation.

Taken together, our study demonstrated that insulin monotherapy reduced the expression of IL-6, a major pro-inflammatory factor involved in vascular damage in T2DM. However, our results also showed that other inflammatory and vascular damage-related factors—specifically RETN, CTSG, and ELANE—remained upregulated. These findings suggested that well controlled T2DM with regular insulin therapy still possess the certain cytokines that could further damage the vascular system, and for patients undergoing long-term insulin therapy, closer monitoring of vascular health may be warranted to mitigate potential complications.

There are several limitations in the study due to the limited sample size and baseline data, ethnic homogeneity, and insufficient in-depth validation of cellular dynamics and interactions occurring within the biological system. However, our study is the first to use high-throughput RNA sequencing to reveal the molecular biological changes brought by insulin from the level of gene expression regulation, and to explain the potential benefits and disadvantages of insulin through bioinformatics analysis, which will provide new ideas and targets for treating T2DM and the application of insulin in the future.

## 5 Conclusion

RNA-Seq reveals molecular changes at the level of gene regulation in T2DM patients treated with insulin, suggesting that insulin therapy is bidirectional - it can not only reduce the level of inflammation but also cause islet B cell damage and vascular complications. The experimental validation shows that the application of insulin is beneficial to relieve inflammation and oxidative stress, hence probably decrease the insulin resistance. Therefore, for patients with T2DM, the benefit of insulin application is certain.

## CRediT authorship contribution statement

**Nan Gao:** Writing – original draft, Visualization, Methodology, Formal analysis, Data curation. **Xiteng Chen:** Writing – original draft, Validation, Funding acquisition, Data curation. **Jun Yang:** Writing – original draft. **Yuanfeng Jiang:** Writing – review & editing. **Shaochong Bu:** Writing – review & editing. **Xiaomei Bai:** Validation. **Zhenyu Kou:** Validation. **Chunjun Li:** Writing – review & editing. **Fang Tian:** Writing – review & editing, Supervision, Project administration, Funding acquisition, Conceptualization.

## Ethics statement

This study was reviewed and approved by Tianjin Medical University Eye Hospital ethics committee, with the approval number: 2022KY(L)-26.

## Data availability

All the raw and processed RNA-seq files in this research have been deposited in the GSA database under accession code HRA007127 (https://ngdc.cncb.ac.cn/gsa-human/).

## Funding

This research was supported by the grant from the Autonomous and open project of Tianjin Key Laboratory of Retinal Functions and Diseases (2022tjswmm003), 10.13039/501100019615Autonomous and open project of Tianjin Key Laboratory of Retinal Functions and Diseases (2022tjswmq002), 10.13039/501100001809National Natural Science Foundation of China (81900846), and Funded by Tianjin Key Medical Discipline (Specialty) Construction Project (TJYXZDXK-037 A).

## Declaration of competing interest

All authors disclosed no relevant relationships.

## Data Availability

I have shared the link to my data in manuscript.
