# Supplementary material for: The duplexity of insulin: The integrated bioinformatics analysis and machine learning identified key genes for type 2 diabetes
Source: Biochem Biophys Rep. 2025 Jun 24;43:102099. doi: 10.1016/j.bbrep.2025.102099 (PMC12242453; doi:10.1016/j.bbrep.2025.102099)
Supplement: Multimedia component 1 [file mmc1.pdf]

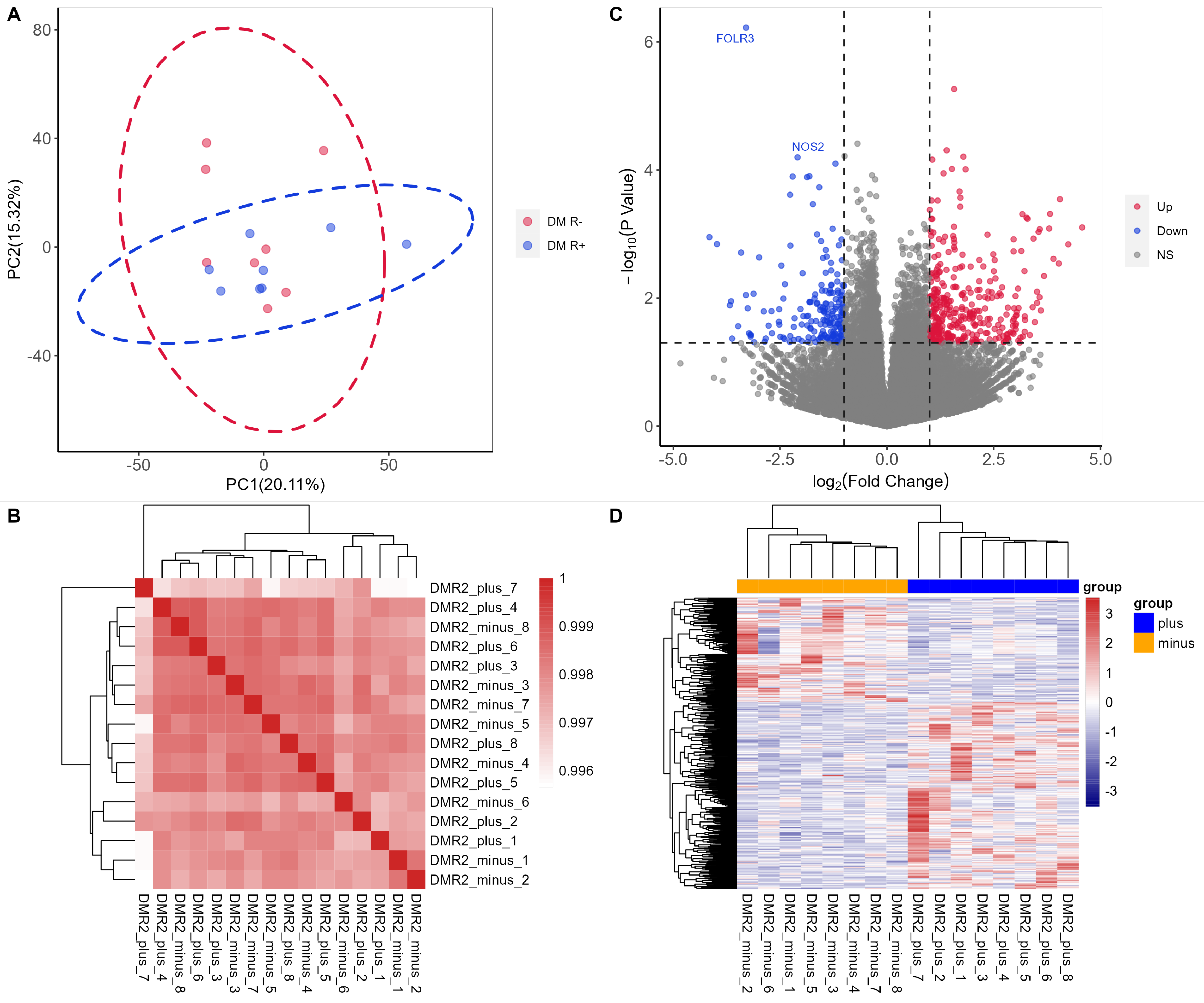
A
80
40
0
-40
-50
0
50
PC2(15.32%)
PC1(20.11%)
DM R-
DM R+
C
FOLR3
NOS2
6
4
2
0
-5.0
-2.5
0.0
2.5
5.0
$-\log_{10}$(P Value)
$\log_2$(Fold Change)
Up
Down
NS
B
DMR2_plus_7
DMR2_plus_4
DMR2_minus_8
DMR2_plus_6
DMR2_plus_3
DMR2_minus_3
DMR2_minus_7
DMR2_minus_5
DMR2_plus_8
DMR2_minus_4
DMR2_plus_5
DMR2_minus_6
DMR2_plus_2
DMR2_plus_1
DMR2_minus_1
DMR2_minus_2
1
0.999
0.998
0.997
0.996
D
group
plus
minus
3
2
1
0
-1
-2
-3
DMR2_minus_2
DMR2_minus_6
DMR2_minus_1
DMR2_minus_5
DMR2_minus_3
DMR2_minus_4
DMR2_minus_7
DMR2_minus_8
DMR2_plus_7
DMR2_plus_2
DMR2_plus_1
DMR2_plus_3
DMR2_plus_4
DMR2_plus_5
DMR2_plus_6
DMR2_plus_8

Fig.S1. RNA-seq data biological repeat quality control and differential analysis. (A) PCA clustering diagram.. The x-label represents the variance contribution rate of principal component 1, and the y-label represents the variance contribution rate of principal component 2. (B) Correlation heatmap. The color of the heatmap indicates the correlation of gene expression levels in the sample, white represents low correlation and red represents high correlation. (C) Volcano plots of RNA-Seq data. Red points represented up-regulated genes in DM R+ group and blue points represented down-regulated genes in DM R+ group. Gray points represented genes with no significant difference. (D) Heatmap of DEGs. Orange represents the DM R+ group and blue represents the DM R- group. Red is high expression and blue is low expression.
