# Supplementary material for: The duplexity of insulin: The integrated bioinformatics analysis and machine learning identified key genes for type 2 diabetes
Source: Biochem Biophys Rep. 2025 Jun 24;43:102099. doi: 10.1016/j.bbrep.2025.102099 (PMC12242453; doi:10.1016/j.bbrep.2025.102099)
Supplement: Multimedia component 2 [file mmc2.pdf]

Table S1 Top five biological process in GO analysis of each cluster.

Cluster	ID	Description	P-value	Gene Count
MCODE1	GO:0030198	extracellular matrix organization	9.31E-07	4
	GO:0043062	extracellular structure organization	9.4E-07	4
	GO:0030199	collagen fibril organization	8.3E-05	2
	GO:0061299	retina vasculature morphogenesis in camera-type eye	0.002912	1
	GO:0038063	collagen-activated tyrosine kinase receptor signaling pathway	0.003177	1
MCODE2	GO:0051873	killing by host of symbiont cells	5.47E-06	2
	GO:0051883	killing of cells in other organism involved in symbiotic interaction	6.37E-06	2
	GO:0050832	defense response to fungus	1.38E-05	2
	GO:0043312	neutrophil degranulation	1.71E-05	3
	GO:0002283	neutrophil activation involved in immune response	1.74E-05	3
MCODE3	GO:0050832	defense response to fungus	0.000128	2
	GO:0001894	tissue homeostasis	0.000139	3
	GO:0009620	response to fungus	0.000223	2
	GO:0019731	antibacterial humoral response	0.000266	2
	GO:0031640	killing of cells of other organism	0.000323	2
