# Supplementary material for: The duplexity of insulin: The integrated bioinformatics analysis and machine learning identified key genes for type 2 diabetes
Source: Biochem Biophys Rep. 2025 Jun 24;43:102099. doi: 10.1016/j.bbrep.2025.102099 (PMC12242453; doi:10.1016/j.bbrep.2025.102099)
Supplement: Multimedia component 3 [file mmc3.pdf]

**Table S2** Top five differential GSVA enrichment pathways.

| Category | Pathway Name | $Log_2$ Fold Change | P-value |
|---|---|---|---|
| KEGG | AMYOTROPHIC_LATERAL_SCLEROSIS_ALS | -0.211 | 0.0057 |
| KEGG | LINOLEIC_ACID_METABOLISM | 0.318 | 0.0104 |
| KEGG | PROTEASOME | -0.358 | 0.0113 |
| KEGG | RIBOSOME | -0.538 | 0.0125 |
| KEGG | PARKINSONS_DISEASE | -0.323 | 0.0127 |
