# Supplementary material for: The duplexity of insulin: The integrated bioinformatics analysis and machine learning identified key genes for type 2 diabetes
Source: Biochem Biophys Rep. 2025 Jun 24;43:102099. doi: 10.1016/j.bbrep.2025.102099 (PMC12242453; doi:10.1016/j.bbrep.2025.102099)
Supplement: Multimedia component 4 [file mmc4.pdf]

Table S3 Top five differential GSVA enrichment pathways.

Category	Pathway Name	Log_2 Fold Change	P-value
KEGG	AMYOTROPHIC_LATERAL_SCLEROSIS_ALS	-0.211	0.0057
KEGG	LINOLEIC_ACID_METABOLISM	0.318	0.0104
KEGG	PROTEASOME	-0.358	0.0113
KEGG	RIBOSOME	-0.538	0.0125
KEGG	PARKINSONS_DISEASE	-0.323	0.0127
